# Superlattice in collapsed graphene wrinkles

**DOI:** 10.1038/s41598-019-46372-9

**Published:** 2019-07-10

**Authors:** Tim Verhagen, Barbara Pacakova, Milan Bousa, Uwe Hübner, Martin Kalbac, Jana Vejpravova, Otakar Frank

**Affiliations:** 1 Department of Condensed Matter Physics, Faculty of Mathematics and Physics, Charles University, Ke Karlovu 5, 121 16 Prague 2, Czech Republic; 2 J. Heyrovsky Institute of Physical Chemistry of the CAS, v.v.i., Dolejskova 3, 182 23 Prague 8, Czech Republic; 3 Faculty of Natural Sciences, Department of Physics, Norwegian University of Science and Technology (NTNU), Høgskoleringen 5, NO-7491 Trondheim, Norway; 4 Leibniz Institute of Photonic Technology (IPHT), PO. Box 100239, D-07702 Jena, Germany; 5 Department of Inorganic Chemistry, Faculty of Science, Charles University, Albertov 6, 128 43 Prague 2, Czech Republic

**Keywords:** Electronic properties and devices, Optical properties and devices

## Abstract

Topographic corrugations, such as wrinkles, are known to introduce diverse physical phenomena that can significantly modify the electrical, optical and chemical properties of two-dimensional materials. This range of assets can be expanded even further when the crystal lattices of the walls of the wrinkle are aligned and form a superlattice, thereby creating a high aspect ratio analogue of a twisted bilayer or multilayer – the so-called twisted wrinkle. Here we present an experimental proof that such twisted wrinkles exist in graphene monolayers on the scale of several micrometres. Combining atomic force microscopy and Raman spectral mapping using a wide range of visible excitation energies, we show that the wrinkles are extremely narrow and their Raman spectra exhibit all the characteristic features of twisted bilayer or multilayer graphene. In light of a recent breakthrough – the superconductivity of a magic-angle graphene bilayer, the collapsed wrinkles represent naturally occurring systems with tuneable collective regimes.

## Introduction

A twist between the individual layers of a graphene bilayer enables the observation of new phenomena, such as superconductivity or Mott insulating states, which are unknown for single layer graphene^1^. These phenomena can be observed, because the layer orientation differs from the perfectly oriented AA or AB stacking, and a superlattice structure is formed^2^. At specific orientations (magic angles), the graphene bilayer exhibits ultra-flat bands near charge neutrality, which upon doping results in zero-resistance states with a critical temperature Tc up to 1.7 K^1^.

Twisted bilayers can be created by stacking exfoliated or chemical vapour deposition (CVD)-grown graphene monolayers^3^, folding a graphene monolayer using an atomic force microscope (AFM)^4,5^ or even directly by growing twisted bilayer graphene using CVD^6^. Twisted bilayer graphene with the misorientation angle in a suitable range can be conveniently identified using Raman spectroscopy, as superlattice-activated Raman processes give rise to two new Raman modes: R_TO_ and R_LO_^4,7,8^, where the subscripts refer to the respective phonon branches. The R_TO_ and R_LO_ modes originate from the phonons with a wave vector ***q*** in the interior from the Brillouin zone. The activated wave vector is dependent on the actual misfit angle *θ*; therefore, the misfit angle can be determined from the measured Raman shift of the R_TO_ and R_LO_ modes. The same Raman features have been found also in systems with more than two twisted graphene layers^9,10^. On the other hand, the reports on twisted bi- or multilayer graphene systems have been so far limited to planar structures, with the notable exception of graphene nanoscrolls^11^.

Out-of-plane corrugations are ubiquitous in graphene due to their low bending stiffness, resulting in various shapes and dimensions: from (sub)nanometre-sized, low aspect ratio ripples to long, high aspect ratio wrinkles^12^. It was theoretically shown that if the wrinkle height is sufficiently large, the wrinkle can collapse^13^ in such a way that the distance between the walls of the wrinkle becomes small enough to effectively form a bilayer^13–17^. If the height of such a wrinkle does not reach approximately 8 nm^13,15,18^, the collapsed wrinkle can remain standing. However, after exceeding the critical height, the wrinkle bends towards the surface and is termed ‘folded collapsed wrinkle’^14^. In such a case, depending on the number of folds, the resulting structure will be composed of 2n + 1 layers (n > 0). Thus, in some cases, it can be expected that both walls of the graphene wrinkle will be oriented in such a way that they form a collapsed wrinkle consisting of a twisted bilayer, if standing, which we call a ‘twisted bilayer wrinkle’, or twisted multilayer wrinkle, if folded. That said, the appearance of twisted wrinkles may not be as rare as could be expected^9,17^. Wrinkles are common to transferred CVD-grown graphene and usually form during the cooling phase as a result of a thermal expansion coefficient mismatch between the metal catalyst and graphene^12,19^. These out-of-plane corrugations usually manifest as long and narrow wrinkles spaced by several micrometres (depending on the particular graphene growth and transfer procedures)^13^. The adhesion of the graphene is high on the standard substrate (Si/SiO_2_)^20^, and thus the compressive stress is partially retained at the interface, and only a part of the layer is relaxed into the wrinkles. However, if such a substrate presents a large number of instabilities (such as height topography corrugations/features), then the probability of the appearance of wrinkles will be higher^21,22^.

The selective formation of wrinkles with a specific geometry has a promising impact on future applications, as the morphology of graphene itself is a factor which plays an important role in its actual band structure. Graphene’s effective gauge field follows the local curvature of the topographical networks of rugae and can change locally the electronic^13^, optical^23^ and chemical properties^24^ of corrugated graphene compared to flat graphene^12,25^. Furthermore, exotic states (such as snake states) are expected to be observable in these thin wrinkles^26^. Wrinkled graphene also exhibits interesting mechanical properties, e.g., an increased bending stiffness of standing collapsed wrinkles^27^ or an improved interfacial shear stress transfer at the graphene/polymer interface when the graphene wrinkles are fully supported by the polymer^28^.

The prospect of combining the electronic, optical and chemical properties of wrinkles and twisted bilayer graphene is very appealing from the perspective of technical realisation, as both outward sides of such a vertical graphene bilayer are easily accessible for creating contacts or for chemical functionalisation. The twisted bilayer and multilayer wrinkles thus have interesting applications in optoelectronics due to their high aspect ratio (length/width) and relative ease of patterning, e.g., by modifying the protocol presented in ref.^29^. From a fundamental point of view, the twisted wrinkles represent a perfect model system for the observation of various collective phenomena, such as superconductivity or Mott insulating regimes.

In the present letter, we demonstrate that the ‘twisted wrinkles’ do indeed exist, proving them via the synergy of atomic force microscopy (AFM) and Raman micro-spectroscopy studies. The AFM measurements showed topographic features characteristic of both narrow standing wrinkles and broad folded ones. Indisputable evidence of the presence of twisted wrinkles was generated by the combination of Raman spectroscopic mapping and Raman spectroscopy co-localised with AFM using a range of visible excitation energies which exhibit the characteristic properties of twisted bilayer graphene, such as the R_TO_ and R_LO_ modes, and the excitation energy dependence of the giant enhancement of the G mode.

## Results and Discussion

**Figure 1:**
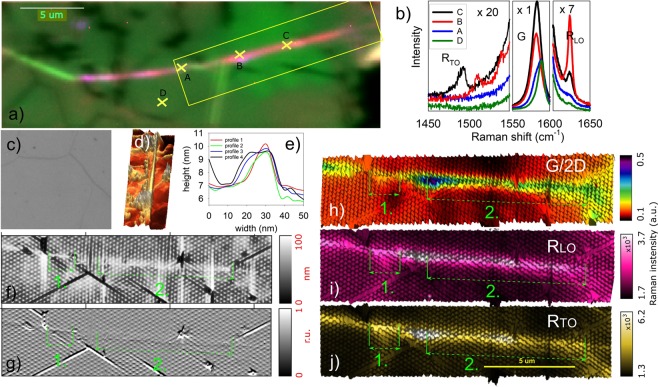
Example of the selected part of the optically visible wrinkle with clearly observable thin/narrow wrinkle parts, visualised by AFM and co-localised with the Raman spectral maps. (**a**) Overlay map of the intensity of the G, R_TO_ and R_LO_ Raman bands. Raman spectra of the G band region in individual points (**b**) are tagged with yellow crosses. (**c**) Optical image of an area with visible wrinkles. (**d**) Magnified 3D AFM topography detail of the thin wrinkle and (**e**) cross-sections perpendicular to its axis. (**f**–**j**) Images of the area highlighted by the yellow rectangle in (**a**): (**f**) AFM topography and (**g**) amplitude, 3D AFM topography image coloured with the Raman G/2D intensity ratio (**h**) and integral intensities of R_LO_ (**i**) and R_TO_ (**j**) modes. Green arrows in (**f**–**j**) label regions ‘1’ and ‘2’ (see text).

Monolayer graphene transferred to the nanopillar arrays showed a large number of wrinkles that could be easily located optically (Fig. 1(c)) thanks to their strong optical contrast^30^. These wrinkles typically exhibit a fine structure, such as shown in Fig. 1(f,g), where AFM topography and amplitude images of the typical optically visible graphene wrinkle on the top of the nanopillar array, propagating over a large distance in the range of several μm, are shown. The cross-section of the wrinkle topography perpendicular to its propagation direction shows a broad profile (from several tens to hundreds of nm) which is significantly narrowed when approaching the reverse end of the wrinkle. Such a profile (the combination of the high narrow wrinkle broadened into the smaller one) can be attributed to the folded collapsed wrinkle with the edge bulging upwards due to curvature limits^13,15^. Furthermore, Fig. 1(f–g) shows that the profile of the folded wrinkle changed along its length, and several different regions can be identified. Examining the wrinkle from left to right, the first section, *ca*. 3-μm long and 200-nm wide, ended with a perpendicular crack; then, the wrinkle continued only as a 1-μm long, narrow, high wrinkle (i.e., without the broad part), labelled as region ‘1’. Another crack perpendicular to the wrinkle propagation direction again caused a change in its morphology, and the wrinkle propagated further to the right as a broad wrinkle (~500 nm width) with a higher narrow profile superimposed at its bottom edge (region ‘2’).

The analysis of the selected cross-sections of the thin wrinkle (Fig. 1(e)) demonstrated that the ratio of wrinkle height to full width at half maximum (FWHM) was in the interval 0.2–0.5. One would expect that the width (or FWHM) of a real twisted wrinkle would be smaller than the width of our thin wrinkle. However, the measured AFM topography represents the convolution of the real surface topography and the AFM tip; hence, the measured width of the wrinkle was affected by the shape of the AFM tip apex (with the FWHM typically 10 nm; see Fig. S1 in the ESI). The FWHM of the narrow wrinkle was approximately between 10 and 20 nm comparing the narrowest and widest wrinkle cross-section profiles, which corresponds to the FWHM of the AFM tip apex. The interlayer distance, *d*, is only 0.34 nm for the AB-stacked bilayer graphene. Even though it has been shown recently that the equilibrium interlayer distance is dependent on the misfit angle and can be as large as 0.6 nm^31^, it is impossible to resolve the real width of the wrinkle in AFM topography even with a very sharp tip. High resolution surface potential measurement can provide clues to distinguish standing and folded collapsed wrinkles on a flat support due to doping effects from the substrate^18^, however, the very same effect could make the interpretation of Raman spectra more difficult, see below.

**Figure 2:**
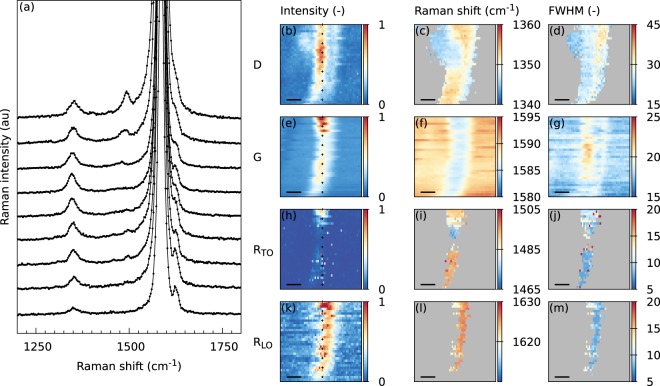
Stacked Raman spectra along the wrinkle, which are offset for clarity. The position of the spectra is indicated in panels (b,e,h,k) with black+. On the right side, Raman spectral maps of the fitted intensity, Raman shift and FWHM are shown for the D-like (**b**–**d**), G (**e**–**g**), R_TO_ (**h**–**j**) and R_LO_ (**k**–**m**) modes. The black scale bar in each map corresponds to 500 nm.

To clarify the nature of the observed wrinkles (Fig. 1(a,b,h,i,j)), we measured the Raman spectra in exactly the same area. Figure S2 (ESI) shows a typical Raman spectrum of the optically visible graphene wrinkle and one reference spectrum of graphene on the nanopillars, measured several μm away from the wrinkle position. The reference spectrum exhibits clearly visible G and 2D modes characteristic of the graphene monolayer, but does not exhibit a D mode. The Raman spectra obtained at the wrinkle location are completely different. The intensity of the G mode is much larger than the intensity of the G mode in the reference spectrum, and a small D-like mode appears in the spectra^32^. Moreover, the FWHM of the 2D mode is larger in comparison with the 2D mode in the reference spectrum; and, finally, the features of a twisted bilayer, the R_LO_ and R_TO_ modes, appear (see detail in Fig. 1(b)). Co-localisation of the captured maps of Raman intensity with the AFM image of the same region (Fig. 1(h–j) and Figs S3–S5 in the ESI) shows that the G mode is indeed significantly enhanced only in the position of the optically visible/narrow wrinkle. Moreover, the R_LO_ mode is selectively enhanced in those regions as well. It must be noted that the 2D band is enhanced only in some parts of the wrinkle, and even in those cases, the enhancement is much lower than in the case of the G band. The significantly larger enhancement of the G mode compared to the 2D mode is well documented by the G/2D intensity ratio in Figs 1(h) and S5 (ESI), where the wrinkle stands out in clear contrast to the ‘flat’ area. We must emphasise that the observed intensity changes are not related to any possible doping changes induced by the substrate, because the graphene was almost completely (95%) suspended. The nanopillars were in contact with only 5% of the graphene, and consequently the substrate-induced Fermi level shift was negligible^21^. The changes in the 2D band intensity can therefore be ascribed solely to the superlattice formation^4^. Figure 1b also shows a downshift of the G band position, more pronounced for the points, where its intensity is increased. The average downshift of the enhanced G band position compared to the non-enhanced one amounts to ~4 cm^−1^ (see also Fig. 2f). The shift is, most probably, a superposition of two effects: (i) altered stress field in the vicinity of the wrinkle^33^, and (ii) interaction of the two layers when they form a bilayer, as observed previously on a large set of twisted bilayers with downshift of ~1–3 cm^−1^ ^6^. We can conclude that the narrow wrinkle indeed exhibits the properties of a twisted bilayer (multilayer) and can henceforth be termed ‘twisted bilayer (multilayer) wrinkle’.

The spatial dependence of the Raman spectra of the twisted wrinkle was studied in detail using a laser energy of 2.33 eV (Fig. 2). Figure 2a shows the Raman spectra along the wrinkle together with the maps of the refined intensity, Raman shift and FWHM for the D-like, G, R_TO_ and R_LO_ modes.

As can be seen in Fig. 2(b), the D-like mode is only visible at the wrinkle location. Furthermore, the intensity of the D-like mode is not constant and becomes larger at the top of the map. This is also clearly visible in the spectra in panel (a). It can be seen that the spectra evolve along the wrinkle when following it from the bottom to the top. The weak R_TO_ mode becomes visible on the left side of the G mode, while the R_LO_ on the right side of the G mode becomes less pronounced, although it is still significant. This can also be seen on the maps corresponding to the R_LO_ and R_TO_ modes. At the bottom of the map, the intensity of the R_TO_ mode is small, but becomes larger at the top of the map. The same behaviour can also be observed for the R_LO_ mode, although the intensity of this mode is much larger.

**Figure 3:**
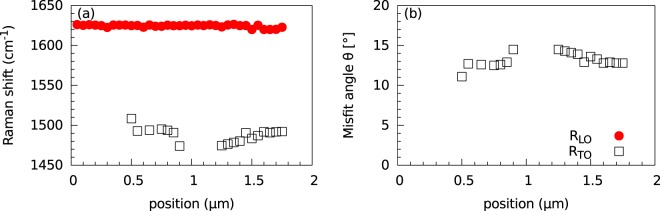
(**a**) Raman shift of the R_LO_ and R_TO_ modes along the wrinkle. The misfit angle from the twisted wrinkle was calculated from the Raman shift of the R_TO_ mode.

The changes in the intensity for the R_TO_ and R_LO_ modes along the wrinkle can be attributed to a slightly different misfit angle due to, for example, a different orientation of graphene grains in the monolayer along the wrinkle or the way the wrinkle was formed. A different misfit angle results in both different Raman shifts of the R modes and a change in their enhancement. Indeed, the change in the Raman shift of the R_TO_ mode is visible in particular (Fig. 2 (i,l)). In Fig. 3(a), these Raman shifts are plotted as a function of the position along the wrinkle, and they show a clear spatial dependence. The R_LO_ mode shows a slow, continuous decrease in wave number. Meanwhile, the R_TO_ mode is not always visible in the spectra, which can be explained by the lower number of data points. The ‘missing’ points around the lateral position of 1 μm suggests changes in the mutual orientation of the wrinkle walls and/or their separation.

The misfit angle *θ* can be derived from the Raman shift of the R_TO_ mode^6,32^ and is shown in Fig. 3(b). For the presented part of the wrinkle, the misfit angle varies between 10° and 15°. In the section between 1.2 and 1.7 μm, the misfit angle continuously decreases, as is also visible for the Raman shift of the R_LO_ mode. Unfortunately, the misfit angle could not be easily calculated from the R_LO_ mode, as the obtained Raman shift was slightly larger than the Raman shift predicted for the R_LO_ mode in the bilayer^6,32^. This may be caused by the slightly modified band structure of graphene due to the wrinkle shape. We also note that the periodicity, *D*, of the moire pattern originating in the bilayer with the above mentioned twist angles, is on the order of 1 nm, according to the formula *D* = *a*/(2*sin(*θ*/2)), where *a* is the graphene lattice parameter^34^. Hence it is obvious that even the height of the standing collapsed wrinkle allows for the creation of a superlattice.

**Figure 4:**
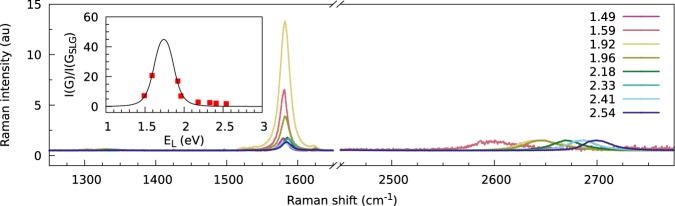
Raman spectra at the wrinkle measured with different excitation energies, normalised to the intensity of the 2D mode. The inset shows the intensity ratio between the G mode at the wrinkle [I(G)] and several μm away from a single layer graphene [I(G_SLG_)] as a function of the laser excitation energy E_L_. Red squares are the measured intensity ratios, and the black continuous line is a least square fit to Eq. 1.

In Figs 4 and S6 (ESI), the evolution of the Raman spectra of the wrinkle with a range of visible excitation energies is shown. It is clearly visible that the intensity of the graphene G mode becomes significantly larger for laser excitations between 1.59 and 1.96 eV. This is also confirmed by comparing the intensity of the G mode of the wrinkle and the monolayer graphene a few μm away from the wrinkle location (inset of Fig. 4). For laser excitations between 1.59 and 1.96 eV, an enhancement factor of almost 20 was achieved. From the excitation profile, the energy difference between the Van Hove singularities (E_VHS_) and the resonance window width (γ) can be obtained from formula^6,32^:

1 $$\frac{I(G)}{I({G}_{SLG})}={|\frac{M}{({E}_{L}-{E}_{VHS}-i\gamma )({E}_{L}-{E}_{VHS}-\hslash {\omega }_{G}-i\gamma )}|}^{2},$$

where *ℏ*ω_G_ is the energy of the G phonon, and *M* is a constant that encompasses the product of the matrix elements for electron-photon and electron-phonon interactions. The inset in Fig. 4 shows the obtained fit of the captured data using the least squares method. For the presented twisted wrinkle, *E*_VHS_ was approximately 1.64 eV, *γ* was 0.14 eV and *M* was 0.20 eV. *E*_VHS_ can then be related to the mismatch rotation angle *θ* using *E*_l_^max^ = *E*_0_|sin(3θ)|^6,32^, where *E*_0_ = 3.9 eV. When *E*_l_^max^ = 1.64 eV, we find that *θ* is approximately 14°. This agrees well with the value of *θ* derived from the position of the R_TO_ mode. We also noted that the giant enhancement of the G mode correlating with the appearance and shifts of the R_TO_ and R_LO_ modes rules out the interpretation of the increased G intensity as a simple addition of graphene layers in the collapsed wrinkle, as proposed previously by Zhu *et al*.^13^. If this were the case, the G band intensity would be roughly multiplied by a factor of three (supposing the collapsed wrinkle forms a trilayer ~700-nm wide, occupying the whole Raman laser spot) and not by a factor of ~20 or more (Figs 2 and 3). Additionally, the 2D band intensity would be multiplied by the same factor, which was not the case.

Figure S6 (ESI) shows the dependence of the Raman intensities and positions of the R modes on the laser excitation energy. Even though the fits of the bands were encumbered by larger errors as their intensities decreased away from the resonance, it is obvious that (i) their intensity followed a similar trend as the G band, and (ii) they did not show a dispersive behaviour. The latter observation stands in contrast to a recent report by Kun *et al*.^25^, who observed a dispersive band at similar wave numbers as the R_LO_ and assigned it to the D’ band. We cannot completely exclude the presence of such a D’ band in our spectra, which would overlap with the R_LO_ mode. However, its intensity would be at least very low.

Finally, to determine whether the G band enhancement originated from standing or folded collapsed twisted wrinkles, we estimated the relative contribution of such a wrinkle to the spectrum in the Gaussian-shaped Raman laser spot. The intensity, *I*, at a given point with coordinates (*x*, *y*) inside the laser spot with radius *r*, centred at (*x*′, *y*′) is given by the following equation^35,36^:

2 $$I(x,\,y)=exp(-2[{(x-x^{\prime} )}^{2}+{(y-y^{\prime} )}^{2}]/{r}^{2}),$$

For the standing wrinkle, we took the theoretical maximal height (before folding down) of 7 nm^15^ and corresponding 100-fold G band intensity enhancement^37^. The laser spot was then divided to 7 × 7 nm^2^ squares, and the Gaussian intensity calculated for each square through Eq. 2. Provided the wrinkle runs through the middle of the laser spot, the sum of squares in the centre line multiplied by the theoretical maximal enhancement factor of 100, divided by the total intensity in the laser spot gives the maximum possible G band enhancement (normalised against I(G_SLG_)) for a standing collapsed wrinkle of ~2.5. No changes in 2D band intensity would be visible in such a case. To achieve the highest G band enhancement observed in this work (50x), the width of the wrinkle running through the middle of the spot must be at least ~200 nm if singly folded according to the calculation above. The observed wrinkle morphologies as well as G band enhancement span the whole range between the borderline cases above. Indeed, the wrinkles with the narrowest AFM profile, i.e., most likely the standing collapsed ones, largely invisible in optical micrographs, show only a minor G band enhancement (Fig. S3); while the broad, folded, collapsed ones, optically visible, provide a large G band enhancement, such as in Fig. 4. However, it is still difficult to discern whether the ‘intermediate’ wrinkles ~50–100-nm wide were simply or multiply folded, since both the height and width profiling were not very accurate in this dimension range.

In conclusion, we have demonstrated the existence of twisted bi- or multilayer wrinkles on CVD-grown graphene transferred to a Si/SiO_2_ pillar array, where the wrinkle walls were aligned with respect to each other. The mutual alignment of the walls creates a graphene superlattice structure, which results in the appearance of the R_LO_ and R_TO_ modes and a laser excitation dependence of the enhancement of the G mode. Furthermore, we found that the mismatch rotation angle slightly varied along the wrinkle owing to the alignment of the wrinkle with respect to the orientation of the graphene domain(s). Both standing and folded wrinkles can be found and discriminated based on their width and G band enhancement factors.

The existence of these twisted wrinkles is important as it presents the opportunity to control the optical and electronic properties of wrinkles in graphene and other 2D materials. For the creation of these well-defined wrinkles, no complex lithographic processes are needed. The formation of wrinkles with a well-defined length/width ratio can be controlled simply via uniaxial compression^29,38,39^ of these materials, by the deposition of 2D materials on predefined substrates^21^, or by deposition of electrodes with a small gap on top of the graphene layer^40^. Furthermore, the interaction between the sidewalls of the wrinkle can be controlled by temperature. The compressive stress, caused by the change of temperature and the negative thermal expansion coefficient of graphene, results in a progressive closing of the wrinkle walls and thus decrease of the interlayer distance^17^. The possibility of fine tuning the level of interaction between two misoriented layers by changing *d* has been shown recently^41^. Finally, the natural superlattices of the wrinkled graphene are promising candidates for facile experimental realisation of high-purity magic angle bilayers, which exhibit tuneable collective states at low temperatures.

## Methods

Graphene samples were grown by the CVD method as reported previously^42^. In brief, the polycrystalline copper foil was heated to 1000 °C and annealed for 20 min under a flow of 50 standard cubic centimetres per minute (sccm) H_2_. The copper foil was exposed to 30 sccm CH_4_ and 50 sccm H_2_ for 20 min, whereafter the copper foil was cooled to room temperature. The as-grown graphene was subsequently transferred onto a Si/SiO_2_ nanopillar array, with a pillar-to-pillar spacing of 240 nm and a pillar top diameter of 54 nm^21^, using nitrocellulose (NC), and residual NC was removed from the graphene by annealing the sample at 180 °C for 30 min in air. Further details are given in the Supporting Information (Fig. S7).

Prepared samples were characterised by AFM and Raman spectroscopy. The Raman spectra were obtained with a WITec alpha300R spectrometer equipped with a piezo stage. Raman spectral maps were measured with 2.33 eV (532 nm) laser excitation, a laser power of approximately 1 mW, a grating of 600 lines/mm and lateral steps of 50 nm in both directions. The laser was focused on the sample with a 100x objective.

The excitation energy-dependent Raman spectra were acquired with a LabRam HR spectrometer (Horiba Jobin–Yvon) using He–Ne [1.96 eV (633 nm)] or Ar^+^–Kr^+^ [2.54 (488), 2.41 (514), 2.33 (532), 2.18 (568), 1.92 (647), 1.59 (785), and 1.49 (830) eV (nm)] lasers. The spectrometer was interfaced to a microscope (Olympus) equipped with a piezo stage, and a line scan was measured through the wrinkle with a 50-nm step size. The intensity response of the CCD detector was calibrated with a tungsten halogen light source (HL-2000-CAL, Ocean Optics) for each excitation energy. The laser was focused on the sample using a 100x objective, and for all wavelengths, a laser power of approximately 1 mW was used.

AFM was measured in the tapping mode with a Dimension Icon microscope (Bruker Inc.). Images were captured using Bruker SCANASYST-AIR probes (k = 0.4 N/m, f_0_ = 70 kHz, nominal tip radius = 2 nm), and the captured topography was processed using the Gwyddion software. Moreover, the co-localised AFM–Raman measurement was performed using the Horiba-AIST-NT Omegascope system (with a LabRAM HR Evolution Raman spectrometer), first capturing the AFM image with the sharp RFESP-300 (k = 40 N/m, f_o_ = 300 kHz) probe by Bruker, followed by the recording of the high-resolution Raman map of the same area using the 532-nm laser excitation wavelength.

## Supplementary information

Supplementary Information

## Data Availability

All data generated or analysed during this study are either included in this published article (and its supplementary information files) or available from the corresponding author on reasonable request.

## References

[1] Cao Y (2018). Unconventional superconductivity in magic-angle graphene superlattices. Nature.

[2] Li G (2010). Observation of Van Hove singularities in twisted graphene layers. Nat. Phys..

[3] Jorio A (2014). Optical-Phonon Resonances with Saddle-Point Excitons in Twisted-Bilayer Graphene. Nano Lett..

[4] Carozo V (2011). Raman Signature of Graphene Superlattices. Nano Lett..

[5] Gupta AK, Tang Y, Crespi VH, Eklund PC (2010). Nondispersive Raman D band activated by well-ordered interlayer interactions in rotationally stacked bilayer graphene. Phys. Rev. B.

[6] Kim K (2012). Raman Spectroscopy Study of Rotated Double-Layer Graphene: Misorientation-Angle Dependence of Electronic Structure. Phys. Rev. Lett..

[7] Jorio A, Cançado LG (2013). Raman spectroscopy of twisted bilayer graphene. Solid State Commun..

[8] Luo Z (2011). Large-Scale Synthesis of Bi-layer Graphene in Strongly Coupled Stacking Order. Adv. Funct. Mater..

[9] Chen X-D (2016). High-Precision Twist-Controlled Bilayer and Trilayer Graphene. Adv. Mater..

[10] Wu J-B (2015). Interface Coupling in Twisted Multilayer Graphene by Resonant Raman Spectroscopy of Layer Breathing Modes. ACS Nano.

[11] Tan P-H (2014). Ultralow-frequency shear modes of 2-4 layer graphene observed in scroll structures at edges. Phys. Rev. B.

[12] Deng S, Berry V (2016). Wrinkled, rippled and crumpled graphene: an overview of formation mechanism, electronic properties, and applications. Mater. Today.

[13] Zhu W (2012). Structure and Electronic Transport in Graphene Wrinkles. Nano Lett..

[14] Zhang K, Arroyo M (2013). Adhesion and friction control localized folding in supported graphene. J. Appl. Phys..

[15] Zhang Y, Wei N, Zhao J, Gong Y, Rabczuk T (2013). Quasi-analytical solution for the stable system of the multi-layer folded graphene wrinkles. J. Appl. Phys..

[16] Kim K (2011). Multiply folded graphene. Phys. Rev. B.

[17] Verhagen T (2017). Tuning the Interlayer Interaction of a Twisted Multilayer Wrinkle With Temperature. Phys. Status Solidi B.

[18] Long F (2016). Characteristic Work Function Variations of Graphene Line Defects. ACS Appl. Mater. Interfaces.

[19] Hattab H (2011). Interplay of Wrinkles, Strain, and Lattice Parameter in Graphene on Iridium. Nano Lett..

[20] Koenig SP, Boddeti NG, Dunn ML, Bunch JS (2011). Ultrastrong adhesion of graphene membranes. Nat. Nanotechnol..

[21] Pacakova B (2017). Mastering the Wrinkling of Self-supported Graphene. Sci. Rep..

[22] Androulidakis C, Koukaras EN, Pastore Carbone MG, Hadjinicolaou M, Galiotis C (2017). Wrinkling formation in simply-supported graphenes under tension and compression loadings. Nanoscale.

[23] Schiefele J, Martin-Moreno L, Guinea F (2016). Faraday effect in rippled graphene: Magneto-optics and random gauge fields. Phys. Rev. B.

[24] Boukhvalov DW, Katsnelson MI (2009). Enhancement of Chemical Activity in Corrugated Graphene. J. Phys. Chem. C.

[25] Kun P (2019). Large intravalley scattering due to pseudo-magnetic fields in crumpled graphene. NPJ 2D Mater Appl..

[26] Oroszlány L, Rakyta P, Kormányos A, Lambert CJ, Cserti J (2008). Theory of snake states in graphene. Phys. Rev. B.

[27] Chacham H (2018). Universal deformation pathways and flexural hardening of nanoscale 2D-material standing folds. Nanotechnology.

[28] Androulidakis C (2017). Wrinkled Few-Layer Graphene as Highly Efficient Load Bearer. ACS Appl. Mater. Interfaces.

[29] Hallam T (2015). Controlled Folding of Graphene: GraFold Printing. Nano Lett..

[30] Campos-Delgado J, Algara-Siller G, Santos CN, Kaiser U, Raskin JP (2013). Twisted Bi-Layer Graphene: Microscopic Rainbows. Small.

[31] Rode JC, Smirnov D, Belke C, Schmidt H, Haug RJ (2017). Twisted Bilayer Graphene: Interlayer Configuration and Magnetotransport Signatures. Ann. Phys. (Berlin).

[32] Carozo V (2013). Resonance effects on the Raman spectra of graphene superlattices. Phys. Rev. B.

[33] Verhagen T, Vales V, Frank O, Kalbac M, Vejpravova J (2017). Temperature-induced strain release via rugae on the nanometer and micrometer scale in graphene monolayer. Carbon.

[34] Kuwabara M, Clarke DR, Smith DA (1990). Anomalous superperiodicity in scanning tunneling microscope images of graphite. Appl. Phys. Lett..

[35] Gupta AK, Russin TJ, Gutiérrez HR, Eklund PC (2009). Probing Graphene Edges via Raman Scattering. Acs Nano.

[36] Li Z (2015). Deformation of Wrinkled Graphene. ACS Nano.

[37] Wu J-B (2014). Resonant Raman spectroscopy of twisted multilayer graphene. Nat. Commun..

[38] Zang J (2013). Multifunctionality and control of the crumpling and unfolding of large-area graphene. Nat. Mater..

[39] Castellanos-Gomez A (2013). Local Strain Engineering in Atomically Thin MoS2. Nano Lett..

[40] Dawood OM (2019). Predicted bandgap opening in highly-oriented wrinkles formed in chemical vapour deposition grown graphene. Mater. Res. Express.

[41] del Corro E (2017). Fine tuning of optical transition energy of twisted bilayer graphene via interlayer distance modulation. Phys. Rev. B.

[42] Kalbac M, Frank O, Kavan L (2012). Effects of Heat Treatment on Raman Spectra of Two-Layer 12C/13C Graphene. Chem. Eur. J..

