# Supplementary material for: Superlattice in collapsed graphene wrinkles
Source: Sci Rep. 2019 Jul 10;9:9972. doi: 10.1038/s41598-019-46372-9 (PMC6620273; doi:10.1038/s41598-019-46372-9)
Supplement: Supplementary file 1 — Supplementary Information [file 41598_2019_46372_MOESM1_ESM.pdf]

Supplementary Material

Superlattice in collapsed graphene wrinkles

Tim Verhagen[1], Barbara Pacakova[2,3], Milan Bousa[2], Uwe Hübner[4], Martin Kalbac[2], Jana Vejpravova[1,5,#], and Otakar Frank[2,]*

[1] Department of Condensed Matter Physics, Faculty of Mathematics and Physics, Charles University, Ke Karlovu 5, 121 16, Prague 2, Czech Republic

[2]J. Heyrovsky Institute of Physical Chemistry of the CAS, v.v.i., Dolejskova 3, 182 23 Prague 8, Czech Republic

[3]Faculty of Natural Sciences, Dept. of Physics, Norwegian University of Science and Technology (NTNU), Høgskoleringen 5, NO-7491 Trondheim, Norway

[4]Leibniz Institute of Photonic Technology (IPHT), PO. Box 100239, D-07702 Jena, Germany

[5]Department of Inorganic Chemistry, Faculty of Science, Charles University, Albertov 6, 128 43, Prague 2, Czech Republic

Corresponding author emails: [*]otakar.frank@jh-inst.cas.cz; [#]jana@mag.mff.cuni.cz

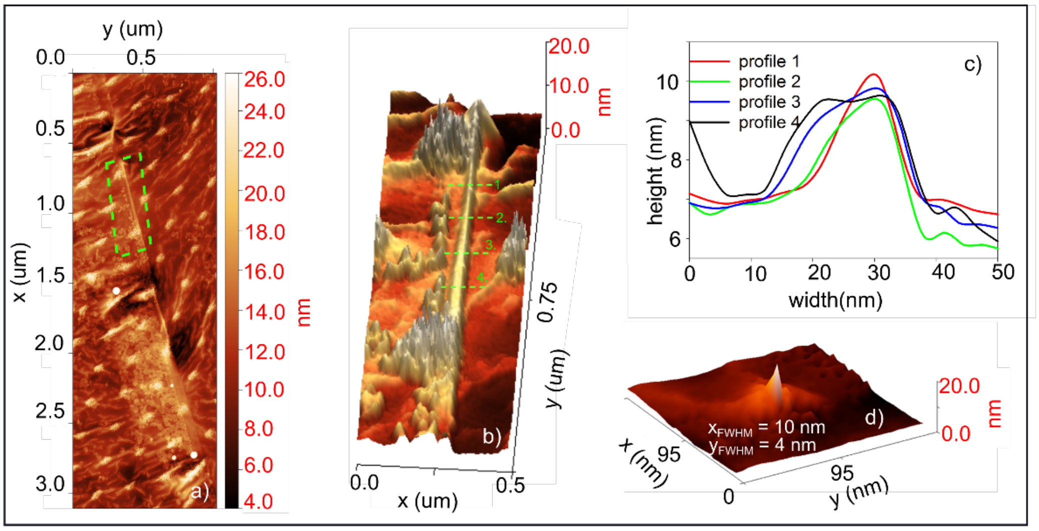

Figure S1. The comparison of the profile of the narrow wrinkle and the AFM tip. (a) The AFM topography image of the narrow wrinkle, with the 3D topography (b) and the wrinkle profile cross sections (c) in selected areas. The narrowest parts of the wrinkle correspond well with the cross-section profile of the AFM probe (d), as a result of the convolution of tip and the narrow wrinkle. It is then expected that the wrinkle is narrower than is its actual profile cross-section width.

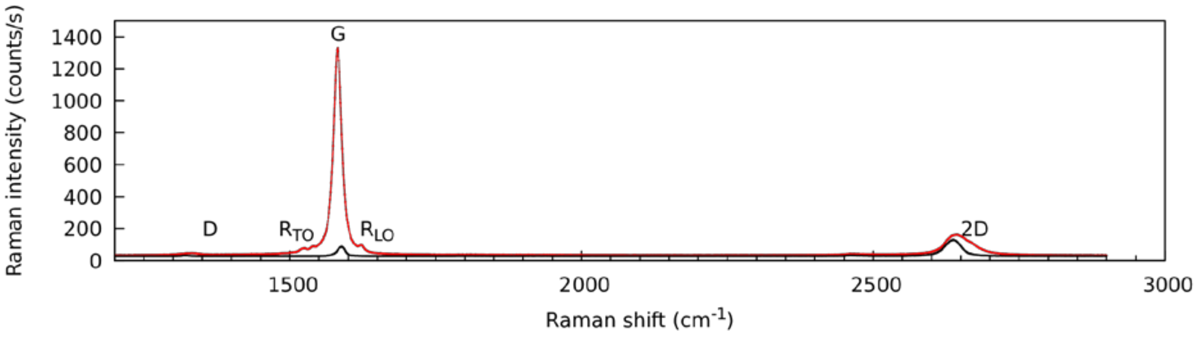

Figure S2. Raman spectrum on the optically visible wrinkle (red) and on ‘flat’ graphene supported by nanopillars (black). Laser excitation energy is 1.92 eV.

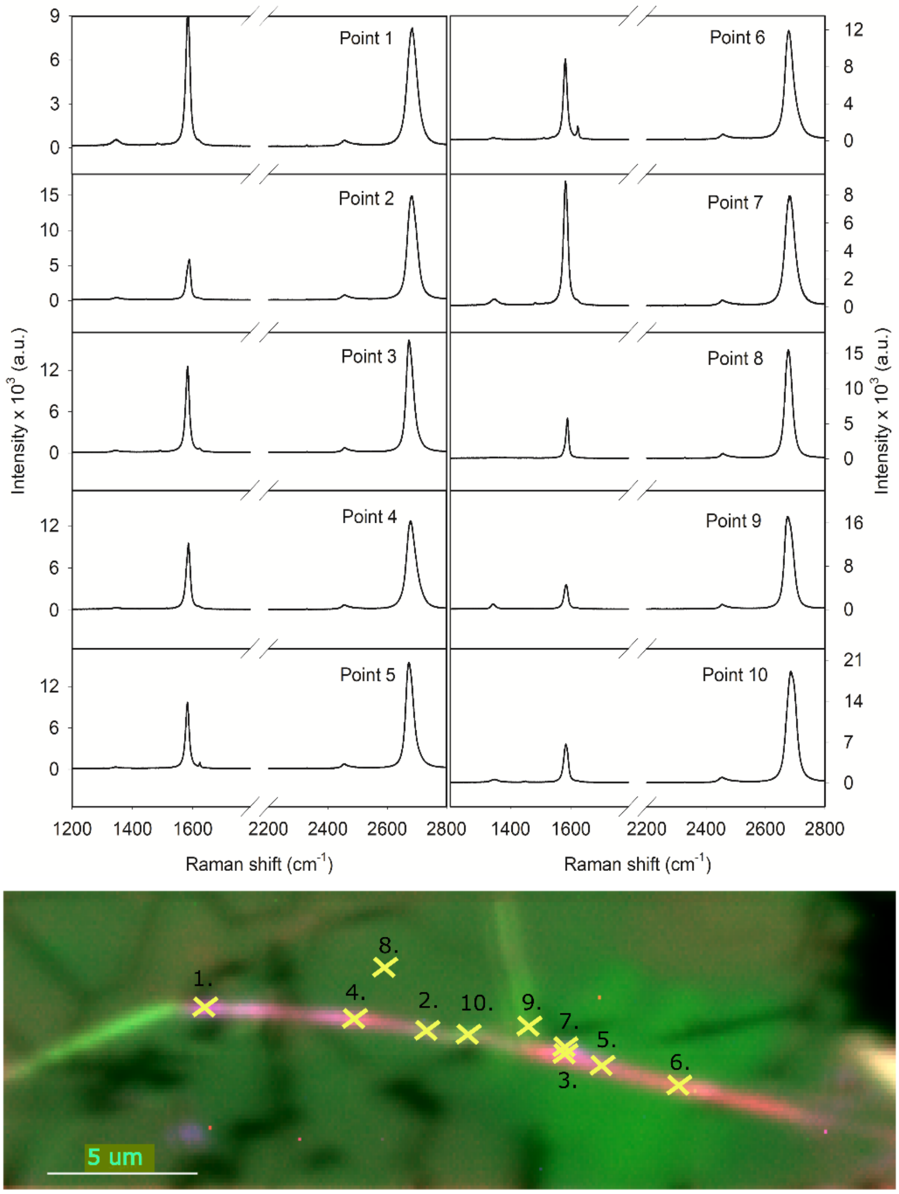

Figure S3. Raman spectra of several points along the wrinkle.

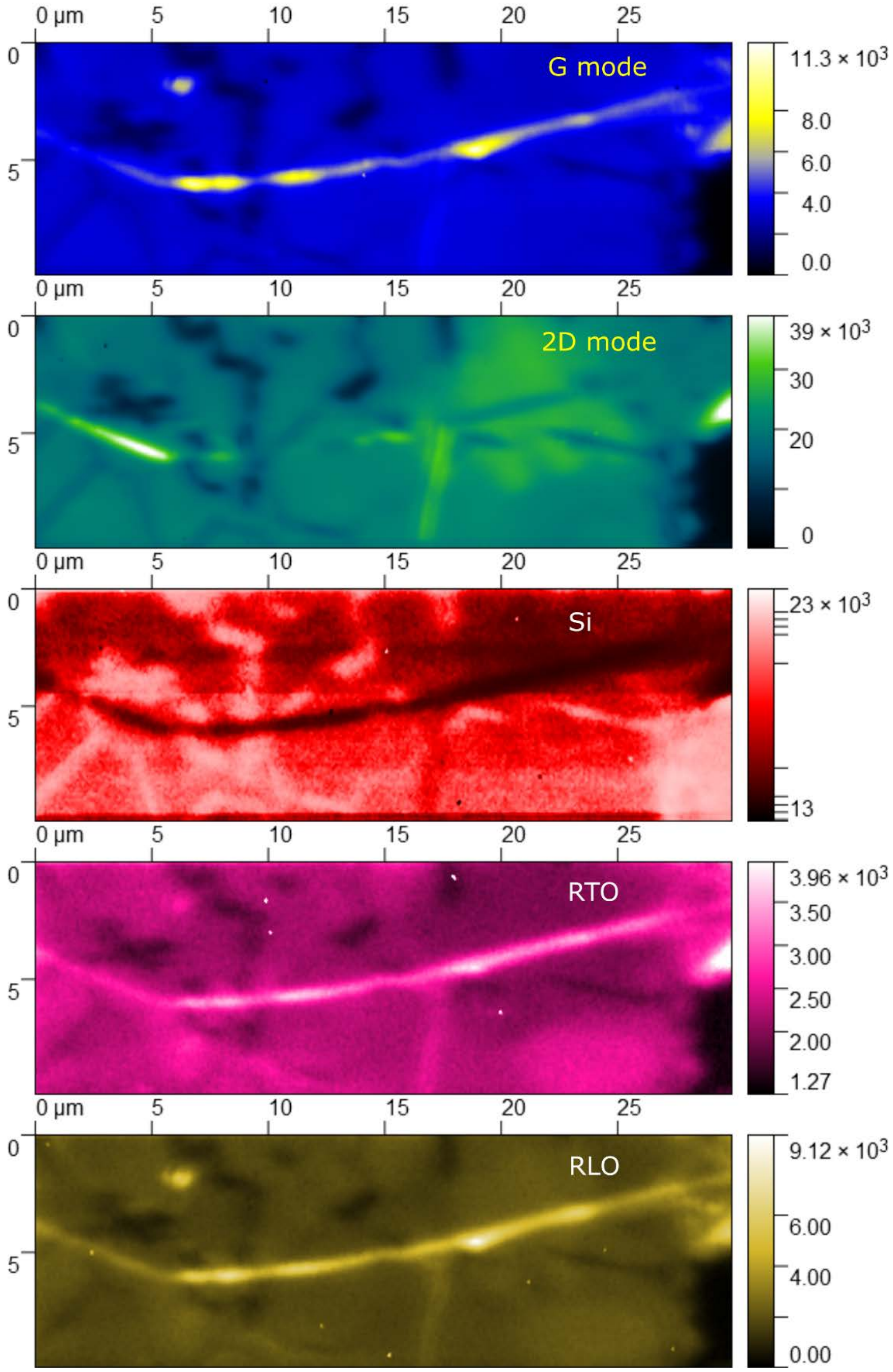

Figure S4. The Raman spectral maps of the optically visible wrinkle with the thin wrinkle at the edge, as was shown in Figure 1(a) in the manuscript body. The maps of integrated intensities for G (a), 2D (b), Si (c), R_{TO} (d), and R_{LO} (e) modes are shown.

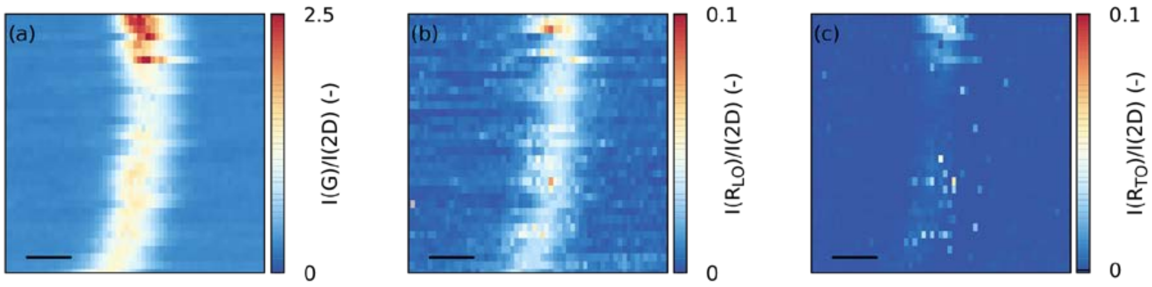

Figure S5. Intensity ratios of the G, R_{LO} and R_{TO} bands with respect to the 2D band. The area corresponds to Fig. 2 in the main text.

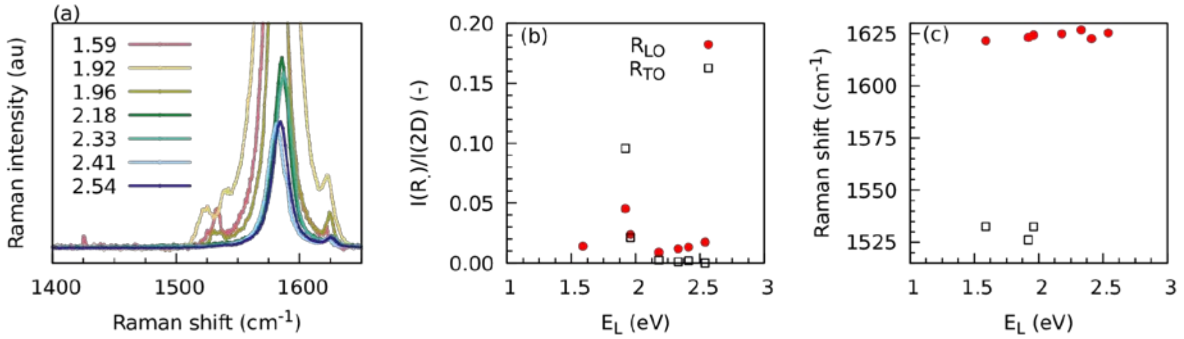

Figure S6. Evolution of the Raman spectra with the excitation energy in the G band region corresponding to Fig. 4 in the main text. The linear relation between the Raman shift of R_{LO} and excitation energy is 3.37 ± 1.90 cm^{-1}/eV, well within the variation of the R_{LO} shift along one wrinkle with single excitation (e.g., Fig. 2, main text).

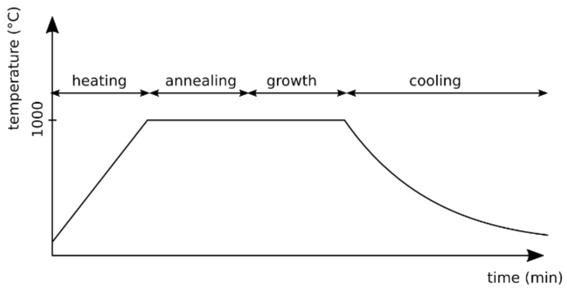

Figure S7. Growth of the CVD graphene. The heating stage to 1000°C proceeds at a rate of 45°C/min, i.e. with the duration of ~21 min. Both the annealing stage and the growth stage durations are 20 min. The cooling is achieved by opening the oven, with 0.05 standard l/min H_2. Base pressure of 350 mTorr was maintained during the whole growth. No specific cleaning or polishing procedure of Cu foil was used before the growth. Copper etchant ce-100 is used after the deposition of the sacrificial polymer on the graphene/Cu stack. After the removal of nitrocellulose at 180°C, the graphene is regularly checked by XPS for the presence of copper, iron and nitrogen to exclude a significant contamination of the processed material.
